# ‘The Mould that Changed the World’: Quantitative and qualitative evaluation of children’s knowledge and motivation for behavioural change following participation in an antimicrobial resistance musical

**DOI:** 10.1371/journal.pone.0240471

**Published:** 2020-10-29

**Authors:** Jennifer Hall, Leah Jones, Gail Robertson, Robin Hiley, Dilip Nathwani, Meghan Rose Perry

**Affiliations:** 1 Regional Infectious Diseases Unit, Western General Hospital, Edinburgh, United Kingdom; 2 British Society of Antimicrobial Chemotherapy, Birmingham, United Kingdom; 3 Public Health England, London, United Kingdom; 4 School of Mathematics, University of Edinburgh, Edinburgh, United Kingdom; 5 Charades Theatre Company, Edinburgh, United Kingdom; 6 University of Dundee, Dundee, United Kingdom; 7 Centre for Immunity, Infection and Evolution, University of Edinburgh, Edinburgh, United Kingdom; 8 Centre for Inflammation Research, University of Edinburgh, Edinburgh, United Kingdom; Università degli Studi di Perugia, ITALY

## Abstract

**Background:**

A primary school musical (“The Mould that Changed the World”) was developed as a unique public engagement strategy to combat antimicrobial resistance (AMR) by engaging children in the story of the discovery of antibiotics, the risks of drug-resistant infections and the importance of prudent antibiotic use.

**Methods:**

The musical intervention was implemented in two UK primary schools by music specialists through a series of workshops, associated learning resources and performances to relatives. Participating children (n = 182), aged 9 to 11 years, were given an online questionnaire in the classroom before rehearsals began and at two weeks post-performance with a six-month evaluation in one school. The impact of the musical was analysed using generalised linear models to control for confounding factors. For the qualitative evaluation, fifteen participating children were selected randomly from each school to take part in semi-structured focus groups (n = 5 per group) before rehearsals began and two weeks post-performance.

**Findings:**

Knowledge gain was demonstrated with children being more likely to answer questions on key messages of the musical correctly at two weeks post- performance (response rate 88%, n = 161) compared with the pre-rehearsal questionnaire (response rate 99%, n = 180) (bacteria can become resistant to antibiotics OR 4.63, C.I. 2.46–9.31 p<0.0001, antibiotic resistant infections can be life threatening OR 3.26 C.I. 1.75–6.32 p = 0.0001, prudent use of antibiotics will slow the rise of antibiotic resistant infections OR 2.16, C.I. 1.39–3.38, p = 0.0006). Long term knowledge gain was demonstrated by a consistent level of correct answers on key messages between two weeks (response rate 95%, n = 89) and 6 months post musical (response rate 71%, n = 67). Following the musical children participating in the focus groups (n = 30) articulated a greater understanding of AMR and the risks of antibiotic overuse. They discussed motivation to minimise personal antibiotic use and influence attitudes to antibiotics in their family and friends.

**Interpretation:**

This study demonstrates that musical theatre can improve both short and long-term knowledge. It demonstrates a hitherto infrequently reported change in attitude and motivation to change behaviour in children at an influential age for health beliefs. This unique public health tool has the potential for high impact particularly if rolled out within national education programmes for primary school aged children.

## Introduction

Antimicrobial resistance (AMR) is global public health crisis [1]. Minimising unnecessary antimicrobial consumption is key; on an individual and a population level antimicrobial exposure leads to increased risk of AMR [2, 3]. Despite public health campaigns [4, 5] in many countries antimicrobial consumption continues to rise [1], with a high proportion of inappropriate prescriptions [6]. It is well recognised that engaging and educating the general public with this issue is vital in order to shape attitudes and change behaviour [7–9].

A recent Wellcome trust survey on public perception of AMR called for communication campaigns to move away from only reporting statistics and to make the ‘issue feel real and relevant’ for the audience [7]. General practitioners report that patient expectations for antibiotics can influence prescribing practices, therefore there is a need to empower and inform the public [10]. Here we introduce a novel public engagement strategy: the use of musical theatre as a tool to educate the general public about antibiotics. The power of anecdote over statistics has been recognised; the emotion that is attached to a story creates a connection to the listener and can have a significant impact on future decision-making behaviour [11]. Theatre has been successfully used as an educational tool in several different spheres, particularly in discouraging smoking, leading healthy lifestyles and improving HIV awareness [12–14]. Health attitudes start at an early age and evidence from climate change educational interventions indicate that children’s behaviour also influences adult behaviour within a household [15].

**Fig 1:**
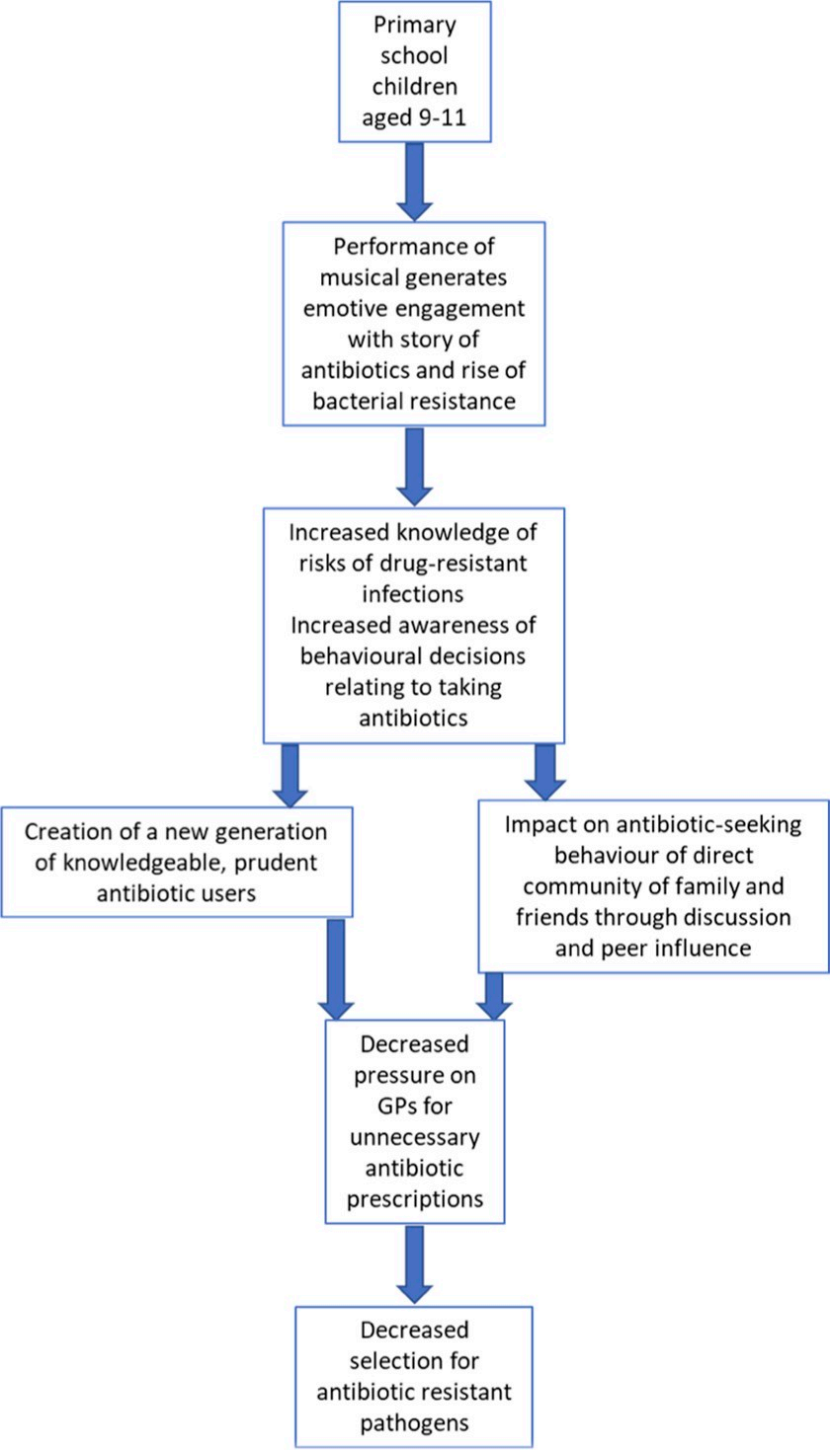
Pathway to impact on antimicrobial resistance through raising awareness in children.

The primary school musical “The Mould that Changed the World” was developed with the explicit aim to educate and engage the public about the importance of conserving antibiotics and the concept of AMR. The key messages of the musical were that bacteria can become resistant to antibiotics, antibiotic-resistant infections can be life threatening and prudent use of antibiotics can slow the rise of AMR. We therefore hypothesised that taking part in the musical would improve knowledge of infectious diseases and AMR, promote careful use of antibiotics and incite intention to encourage the same behaviour in direct contacts (Fig 1).

This paper outlines the quantitative and qualitative evaluation of the impact of this musical theatre intervention in two primary schools when used as a public education tool.

## Methods

### Musical development

The primary school musical “The Mould that Changed the World” was developed in close collaboration between a composer (RH) and an infectious diseases doctor (MRP) with the aim of creating a work that was educational but also enjoyable and engaging, providing a positive holistic learning experience for children. It follows the discovery of penicillin through the life of Alexander Fleming and extends beyond to modern antibiotic use and the rise of resistance [16, 17] and is historically and scientifically accurate. The cast is made up of three classes forming three main character groups: bacteria, soldiers/patients and scientists/doctors. The musical contains songs specific to each character group alongside shared songs sung by two or three character groups together. There are small solo parts within several scenes for a small number of pupils as selected by the teachers. Three adult music and drama specialists perform alongside the children to narrate the show and offer support during the performance by leading in song and movement. The running time of the musical is around 50 minutes.

The musical was designed to map to the National Curriculum in England and the Curriculum for Excellence in Scotland [18, 19]. Although AMR is not specifically named in the curricula, the themes addressed in the musical and the associated resources act as a vehicle for delivery of teaching in a number of different curriculum areas (S1 Fig).

### Musical implementation

A wide number of primary schools around Edinburgh and in London were approached by telephone to offer participation. The first school in Scotland and the first school in England that agreed to participation were selected to become the pilot schools for the project. Participating children in the Scottish school were 9 to 11 years in age whilst pupils in the English school were aged 10 to 11 years old. The musical was delivered at no cost to the school.

Three workshops were delivered in the schools by music and drama specialists. In addition, schools were provided with a bank of learning resources to support further independent work on the musical and associated themes between workshops. These resources included classroom exercises designed to enhance the children’s ability to relate to the content, themes and messages of the musical, as well as musical resources which included lyric sheets, recordings of songs, backing music and score. These resources were hosted online and schools provided with a login that could be shared with all participating children. Further details around the content and timing of these workshops is included in the S1 Table.

The workshops were followed by a daytime final performance by each school. This took place in performance space donated by the National Museum of Scotland in Edinburgh and the Science Museum in London.

### Quantitative data collection

Quantitative data were collected through an online questionnaire which took 10 to 15 minutes to complete (S1 File). Children were asked to estimate how many courses of antibiotics they had taken in their life as a proxy for exposure and where, if ever, they had heard of AMR before to represent prior knowledge. This was followed by 32 questions within five stems which assessed a broad range of infectious diseases knowledge, with a mandatory answer of true or false for each (Table 1). The questions were divided into five categories: i) key messages of the musical; ii) key messages on use of antibiotics iii) themes addressed in the children’s lyrics; iv) themes addressed in the soloist’s lyrics; and v) topics covered in accompanying learning resources only. The key questions were distributed evenly throughout the questionnaire. The same questions were delivered before rehearsals for the musical started and two weeks after the musical performance. The questionnaire was also undertaken six months after the musical in Scotland only due to logistics of pupils having graduated to diverse secondary school placements in England. The 6-month post musical questionnaire had additional free text evaluation questions of “How has the musical changed your view of antibiotics”, “What do you think your role is in this crisis of antibiotic resistance” and “Tell us anything you feel you would like to about what you took away from performing the musical”.

**Table 1:** True/false questionnaire responses from participating children pre-musical and post-musical.

	English school (% correct)		Scottish school (% correct)			Combined (% correct)		% difference
Key messages of the musical	pre-musical	2 weeks post	pre-musical	2 weeks post	6 mths post	pre-musical	2 weeks post	
*TRUE*: *Bacteria can become resistant to antibiotics*	67.5	87.5	75.3	95.5	89.6	**71.7**	**91.9**	**20.3**
*TRUE*: *Antibiotic resistant infections can be life-threatening*	75.9	87.5	75.3	91.0	92.5	**75.6**	**89.4**	**13.9**
*FALSE*: *The number of antibiotic resistant infections in the world is increased by careful*, *prudent use of antibiotics*	37.3	65.3	32.0	41.6	49.3	**34.4**	**52.2**	**17.7**
**Key messages on use of antibiotics**								
*FALSE*: *Infections can only get better if treated with antibiotics*	47.0	69.4	54.6	65.2	74.6	**51.1**	**67.1**	**16.0**
*TRUE*: *Antibiotics are essential for treating serious bacterial infections*	69.9	73.6	83.5	85.4	82.1	**77.2**	**80.1**	**2.9**
*FALSE*: *If I get a headache and runny nose I will definitely need antibiotics*	62.7	66.7	88.7	93.3	97.0	**76.7**	**81.4**	**4.7**
*TRUE*: *If I get a fever it is possible to get better without antibiotics*.	65.1	73.6	73.2	83.1	80.6	**69.4**	**78.9**	**9.4**
**Other concepts referred to in lyrics sung by children**								
*TRUE*: *Infectious diseases can be caused by bacteria and viruses*	90.4	95.8	96.9	95.5	94.0	**93.9**	**95.7**	**1.8**
*TRUE*: *Infectious disease can be spread from person to person*	85.5	90.3	79.4	84.3	79.1	**82.2**	**87.0**	**4.7**
*FALSE Hand washing does not help in reducing transmission of infectious diseases*	71.1	79.2	73.2	80.9	88.1	**72.2**	**80.1**	**7.9**
*TRUE*: *Self-limiting infections can be fought by the body’s natural defences alone*	47.0	59.7	63.9	64.0	73.1	**56.1**	**62.1**	**6.0**
*FALSE*: *The invention of antibiotics has shortened the average duration of life*	65.1	54.2	68.0	76.4	77.6	**66.7**	**66.5**	**-0.2**
*FALSE*: *Antibiotics can kill viruses*	68.7	54.2	68.0	70.8	61.2	**68.3**	**63.4**	**-5.0**
*FALSE*: *You can take antibiotics without a doctor’s prescription*	68.7	61.1	81.4	71.9	77.6	**75.6**	**67.1**	**-8.5**
*FALSE*: *Antibiotics are cheap and easy to invent*	71.1	76.4	75.3	78.7	85.1	**73.3**	**77.6**	**4.3**
*FALSE*: *Antibiotic resistance means you are resistant to the antibiotics*	57.8	51.4	41.2	49.4	37.3	**48.9**	**50.3**	**1.4**
**Other concepts referred to in lyrics sung by soloists**								
*FALSE*: *All bacteria cause disease*	78.3	87.5	86.6	92.1	91.0	**82.8**	**90.1**	**7.3**
*TRUE*: *Bacteria can live naturally on/in your body and in the environment*	88.0	90.3	96.9	87.6	97.0	**92.8**	**88.8**	**-4.0**
*TRUE*: *Antibiotics were invented less than 100 years ago*	63.9	65.3	68.0	74.2	68.7	**66.1**	**70.2**	**4.1**
*FALSE*: *Antibiotics only kill bad bacteria*	56.6	70.8	53.6	59.6	62.7	**55.0**	**64.6**	**9.6**
*TRUE*: *Antibiotics can disrupt the bacterial ecosystem in your body*	55.4	76.4	64.9	73.0	85.1	**60.6**	**74.5**	**14.0**
**Other concepts addressed in learning resources only**								
*TRUE*: *Certain infections can be prevented by vaccines*	63.9	70.8	83.5	84.3	89.6	**74.4**	**78.3**	**3.8**
*FALSE*: *Antibiotic resistant bacteria cannot spread from person to person*	62.7	61.1	53.6	64.0	91.0	**57.8**	**62.7**	**5.0**
*TRUE*: *Bacteria can pass the ability to be resistant to other bacteria by sharing genetic information*	68.7	62.5	61.9	74.2	97.0	**65.0**	**68.9**	**3.9**
*TRUE*: *Antibiotics can prevent an infection developing following major surgery*	57.8	75.0	77.3	78.7	82.1	**68.3**	**77.0**	**8.7**
*FALSE*: *Antibiotics have no side–effects*	66.3	68.1	80.4	80.9	62.7	**73.9**	**75.2**	**1.3**
*TRUE*: *Anyone can suffer from an antibiotic resistant infection*	78.3	76.4	85.6	82.0	85.1	**82.2**	**79.5**	**-2.7**
*FALSE*: *If I am not given antibiotics by the doctor*, *this means the doctor does not believe I am sick*	69.9	69.4	73.2	73.0	82.0	**71.6**	**71.4**	**-0.2**

### Quantitative data analysis

Quantitative data from the questionnaires were collected using Google Documents and analysed using the statistical program R [20].

The impact of the musical on children’s knowledge of antibiotic resistance was analysed using generalised linear models (GLMs) with binomial error distributions using whether or not each question was answered correctly by the pupil as the response variable. We used these models to examine whether pupils’ responses to each question differed pre- and post-musical, accounting for potential confounding factors that could have affected their ability to answer the question correctly. These included school year (P5, P6, or P7), school attended (Scottish or English school), previous exposure to antibiotics (0, 1 to 5, 5 to 10, >10 times), previous knowledge of antibiotic resistance (none or some). We identified which variables explained a significant amount of variation in correct answers by comparing models including and excluding each variable using likelihood ratio tests (using the ‘Anova’ function in the ‘car’ package in R) [21]. The final model fit was checked by calculating the area under the receiving operator characteristic curve (AUC). Results are reported as Odds Ratios (ORs), adjusted for any confounding factors, of how much more likely the children were to answer question correctly pre or post-musical.

### Qualitative data collection

Qualitative data were collected from children regarding knowledge, attitudes and behaviours around antibiotics, AMR and the musical itself. The Theoretical Domains Framework (TDF) was chosen as a comprehensive framework to guide exploration of behaviour and its determinants [22, 23], from which semi-structured topic guides were developed (LFJ, reviewed by MRP and JNH; S2 File). Qualitative data were collected from a sample of 29 children by researchers (MRP, JNH); 15 from the Scottish School and 14 from the English School. These consisted of the same children before and after the musical with participants selected by the class teacher in a purposive manner. Focus groups consisted of the researcher (MRP and/or JNH) with four or five children and lasted around 15 minutes each, at which point data saturation became apparent. Only participants were present. In addition, brief informal interviews were carried out with teachers by MRP/JNH before and after the musical to seek feedback on the musical process. MRP and JNH are doctors with a clinical interest in Infectious Diseases and received prior training in use of the questioning schedules by LFJ, a health psychologist. Neither MRP nor JNH had any prior relationship with the participating schools nor the pupils participating in the focus groups.

Brief field notes were taken by researchers. Focus groups were audio-recorded with participant and parent/guardian permission. These recordings were transcribed by an independent transcribing service before review by the researchers to verify accuracy prior to analysis.

### Qualitative data analysis

Qualitative data were collated and analysed (JNH) using QSR NVivo 11 software in consultation with psychologist (LFJ) to agree a consensus. An inductive thematic approach was used to analyse transcripts as the emergent nature of deduction is more suited to exploration and discovery [24]. Following this a deductive approach was used by placing key themes from the organic framework into the domains of the TDF to identify important behavioural determinants [22, 23, 25, 26]. The results obtained were reviewed in the context of the key messages set out in the musical and the COM-B model [27] as a framework to understand how the intervention might impact behaviour.

### Ethics

The University of Edinburgh research governance department reviewed the study and confirmed that as it was an evaluation of an intervention it did not require Research Ethics Committee review. Written consent for participation was provided by the parent or guardian of participating children.

## Results

### Quantitative data

One hundred and eighty-two primary school children aged 9 to 11 took part in the musical premieres in the two schools; 94 in Scotland and 88 in England. None of the school children invited to take part declined participation.

### Online knowledge questionnaire

One hundred and eighty children completed an online knowledge questionnaire in a classroom setting with a 99% response rate prior to rehearsals starting for the musical and a response rate 88% two weeks following the performance. For the six month follow up, responses were evaluated in 71% (n = 67) of the Scottish children who performed the musical.

All of the children had covered microbes in their school lessons prior to the musical and the majority of children (112/180, 62%) had heard of AMR. Children reported varying previous antibiotic use with only 29% (n = 52/180) reporting never having received antibiotics.

**Fig 2:**
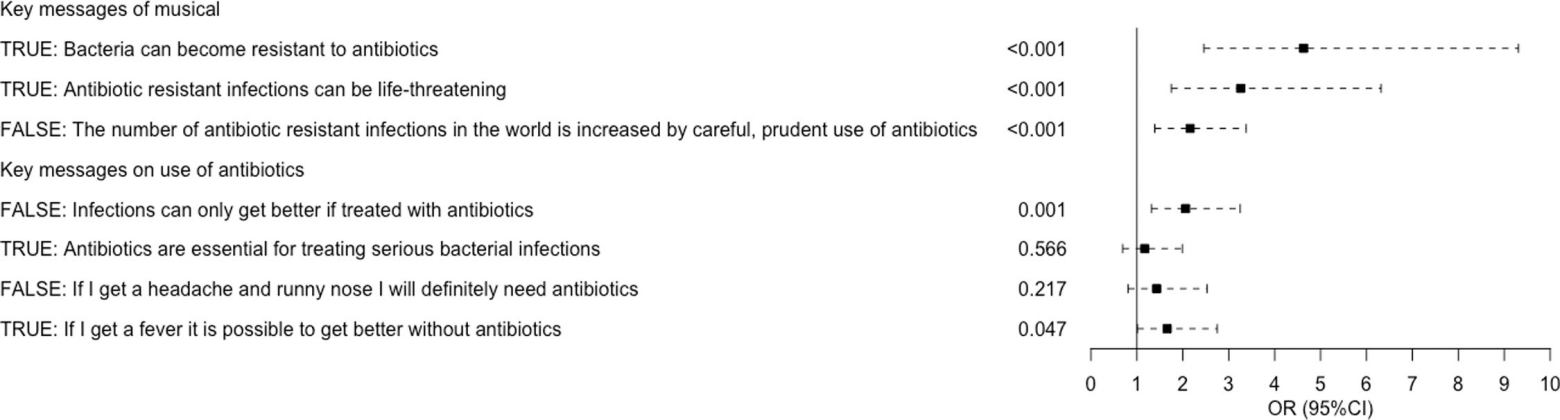
Improvement in children’s knowledge pre- musical and two weeks post-musical. Impact of musical on knowledge in primary school children (n = 161) using a generalised linear model accounting for confounding factors of prior knowledge, exposure to antibiotics, school year and school attended. Prior knowledge of AMR was a significant confounding factor in questions “Antibiotic resistant infections can be life-threatening”, “Infections can only get better if treated with antibiotics” and “If I get a headache and runny nose I will definitely need antibiotics”. School attended was a significant confounder in questions “The number of antibiotic resistant infections in the world is increased by careful, prudent use of antibiotics”, “Antibiotics are essential for treating serious bacterial infections” and “If I get a headache and runny nose I will definitely need antibiotics”.

Using a generalised linear model to account for confounding factors of prior knowledge, exposure to antibiotics, school year and school attended, there was a significant increase in knowledge post-musical in five out of the seven key questions (Fig 2). This demonstrates the children’s increased understanding of the key messages that bacteria can become resistant to antibiotics, antibiotic resistant infections can be life threatening and that prudent use of antibiotics can slow the rise of AMR. Prior knowledge of AMR and school attended were significant confounding factors for some questions.

Of the remaining questions sung in children’s lyrics, soloists’ lyrics and covered in learning resources correct responses at two weeks post musical increased for 16 out of 25 questions (Table 1). This was significant in our model for the questions “not all bacteria cause disease” (OR = 1.9 (95% CIs = 1.3–6.9), p<0.05), “antibiotics can disrupt the bacterial ecosystem in your body” (OR = 2.0 (95% CIs = 1.26–16.4), p<0.01) and “antibiotics can prevent an infection developing following major surgery” (OR 1.6 (95% CIs = 1.2–7.2) p<0.05).

In the online questionnaire two weeks after the musical, 61% (98/161) of children agreed or strongly agreed that the musical had changed the way they viewed antibiotics and 65% (107/161) had enjoyed the experience of performing. Only 13% (21/161) had not found the musical easy to understand.

As our Scottish dataset had longitudinal data we were able to compare pupil responses to key messages at two weeks and six months post-musical using the same generalised linear model. No significant differences in pupil responses were seen confirming that knowledge gain was sustained over this extended period of time. In addition, by including an interaction term in the generalised linear model on the Scottish dataset, we could demonstrate that there was no effect of age on sustained knowledge. This confirms that the musical has positive learning outcomes throughout the age range of 9–11 years in upper primary school.

#### Additional attitude based online questions

In the questionnaire carried out six months after the musical children were given free text to answer the question ‘how has the musical changed your view of antibiotics?’ Fifty three percent (36/67) of answers were positive and included responses such as ‘it makes me and my family not take antibiotics if we have colds, fevers’. In answer to ‘what do you think is your role in this crisis of antibiotic resistance?’, 54% (36/67) of the children responded with answers stating their role was either ensuring that antibiotics are not used too much and/or their role was to spread the message of AMR.

### Qualitative data

#### Knowledge of antibiotics and antimicrobial resistance

Before the musical, focus group children varied in their knowledge about infections, antibiotics and AMR. Knowledge in some areas was limited, for example the widespread belief that antibiotics can treat viral infections. Furthermore, a minority of children thought that antibiotics could treat non-infective conditions such as anxiety or were addictive. Before the musical some children had not heard of AMR. Others had better understanding of AMR, though some of these believed AMR was due to an individual becoming resistant to antibiotics, rather than bacteria. There was disparity in children’s beliefs around the consequences of AMR, with many thinking they would never be affected by AMR whilst others recognised potentially devastating effects (Table 2).

**Table 2:** Selected focus group (n = 29) responses from participating children pre- and two weeks post-musical, by key emerging theme and mapped to the theoretical domains framework.

	Pre-musical	Post-musical
**Gain in knowledge and understanding of antibiotics and AMR**		
**Antibiotics**	*‘I know antibiotics is a medicine for viruses that you have*.*’*	*‘Don’t use antibiotics when you have a cold*.*’*
	(Domain I: Knowledge)	(Domain I: Knowledge)
	*‘I’m thinking that*, *so my mum……*.*I think she takes these anti*, *antibiotics really to calm herself down*.*’*	
	(Domain I: Knowledge)	
	*Child 1*: *‘…if you take more antibiotics than you need …’*	
	*Child 2*: *‘Might get addicted*.*’*	
	(Domain I: Knowledge)	
**AMR**	*‘Is it [AMR] like*, *where bacteria gets too strong or something*, *and the antibiotics can't help it*?*’*	*‘Because bacteria evolved to not*, *so that*, *they can’t*, *the antibiotics can’t stop them*.*’*
	(Domain I: Knowledge)	
	*‘Then won’t be able to take them [antibiotics] anymore because then you’ll be resistant to them*.*’*	(Domain I: Knowledge)
		*‘My takeaway is that we shouldn’t be using the antibiotics when we don’t need it*, *that’s why these bacteria*, *they're getting resistant to it*.*’*
	(Domain I: Knowledge)	
		(Domain I: Knowledge)
**AMR and the future**	*‘… if we just don't take care or we just leave antibiotics*, *more people might just start dying*.*’*	*‘I’ve never taken antibiotics so it’s not going to affect me*.*’*
	(Domain VI: Beliefs about Consequences)	(Domain VI: Beliefs about Consequences)
	*Child 1*: *‘They’ll be something newer and better*.*’*	*Child 1*: *‘Everybody will die*, *the world*, *sorry–'*
	*Child 2*: *‘Yeah they might try to think of a new way to try and create a new medicine using the ones that you already have or just make a new one*.*’*	
		*Child 2*: *‘Loads of people will probably die of infection or other stuff*.*’*
	(Domain VI: Beliefs about Consequences)	
		(Domain VI: Beliefs about Consequences)
		*‘Yeah they might try to think of a new way to try and create a new medicine using the ones that you already have or just make a new one*.*’*
		(Domain VI: Beliefs about Consequences)
		*‘In the future they’ll invent*, *probably they’ll invent something that can cure anything*.*’*
		(Domain III: Social and Professional Role and Identity)
**Motivation to change behaviour in relation to antibiotic use**		
**Reduction in antibiotic use and antimicrobial stewardship**	*‘Maybe we could try and like not take so many antibiotics and our body be able to fight the diseases*, *because then we wouldn’t need antibiotics*.*’*	*‘…if like your mum says you might need to get some antibiotics you might think like maybe I actually probably don’t need that*, *like you could say to your mum I probably won’t need antibiotics for this*.*’*
	(Domain III: Social and Professional Role and Identity)	
	*‘I probably wouldn’t have antibiotics anyway because every time I have a swallow pill I always chew it up and it’s really disgusting*.*’*	(Domain III: Social and Professional Role and Identity)
		*‘Yeah*, *to use them [antibiotics] much more carefully*, *like when you grow up you don’t just pick it because your head’s hurting*.*’*
	(Domain VI: Beliefs about Consequences)	
		(Domain VIII: Intentions)
		*Interviewer*: *Do you think there’s anything that you can do or that we can do to prevent antibiotic resistance*?
		*Child*: *To not overuse it*.
		*Child*: *Be more careful with it and just listen to your doctor’s advice*.
		(Domain III: Social and Professional Role and Identity)
		*‘You shouldn’t overuse them*. *If we have to use them then we have to use the full dose*.
		(Domain III: Social and Professional Role and Identity)
**Motivation to spread the message of prudent antibiotic use**		
**Discussion of AMR with direct community**	*‘My brothers don’t really know about anything about antibiotics and neither do I*.*’*	*‘And so my family really never*, *we never used to talk about it*, *never used to think about it*, *and so I think when people will see this play or hear about it or the message which is coming through*, *I think it’s important that the people who are watching the play*, *that they get the message as well*, *so that it can spread*. *And I think if that happens maybe it might make a difference*.*’*
	(Domain XII: Social Influences)	
	*‘I didn’t really know much about it and I knew what it was but I didn’t really know much about it and I’ve not really mentioned*, *asked my parents about it so I don’t know if they know or not*.*’*	
	(Domain XII: Social Influences)	
		(Domain VI: Beliefs about Consequences)
**Spreading the message wider**		*‘Because spreading the message*, *it feels like you’re a good person*, *like you want to make the world think*, *yeah*.*’*
		(Domain IX: Goals)
		*Interviewer*: *‘And what do you think is your role personally to make a difference to antibiotic resistance*?*’*
		*Child*: *‘To warn people who are*, *who you might think are using* [antibiotics] *the wrong way*.*’*
		(Domain VIII: Intentions)
**Evaluation of musical as an educational technique**		
**Opinions on experience**	*‘I think [we’ll] learn quite a bit too because if in the musical you’re telling facts in that kind of way then it might be quite*, *it would be a bit more interesting than just reading them online or something*.*’*	*‘Because it’s a bit boring if an adult just comes and stands in front of the class and goes blah*, *blah*, *blah*, *that’s that*. *Whereas you*, *when you do a musical and you dress up and you learn the songs*, *it puts yourself in the place of whoever you are and so you understand more*.*’*
		*‘And because we were so excited at being able to perform in a special space*, *we tell our families and stuff that we get to perform in the RI theatre [the Science Museum] and then when you tell them that they’ll*, *you also will tell them the meaning and then they’ll tell other people and spread faster*.*’*
		*Interviewer*: *‘And how did performing the musical make you feel*?*’*
		*Child 1*: *‘Really happy*.*’*
		*Child 2*: *‘And proud*.*’*
		*Child 3*: *‘Very happy and excited and yeah*, *proud of ourselves–'*
		*Child 4*: *‘I felt like I have achieved so much at the end–'*

These polarised views around the potential consequences of AMR persisted after the musical. The individuals who felt they would not be affected demonstrated faith in science and technology to develop new antibiotics and new ways of treating infection, or felt they would not be affected because they have never taken antibiotics in the past. Despite this, after the musical children demonstrated more consistent and improved understanding of antibiotics as well AMR and the factors contributing to this, crucially antibiotic overuse (Table 2).

#### Behaviour change in relation to infectious diseases and antibiotics

Before the musical, some children were able to identify the need to reduce antibiotic use to reduce AMR. However, none reported intention to change behaviour in future because of this, with a minority citing other reasons to reduce antibiotic use such as taste (Table 2).

In contrast, following the musical many children cited motivation to spread awareness of AMR and change behaviour, with a shift from suggesting vague solutions such as ‘technology’ to more specific examples of antimicrobial stewardship such as reducing our individual antibiotic use, engaging with medical professionals and taking antibiotics as prescribed (Table 2).

This suggests that, as well as improved knowledge, participation in the musical also led children to recognise ways to change their own behaviours and feel empowered in their health behaviours around antibiotics.

#### Engagement of children to spread the message of AMR

Before the musical, children reported that AMR was not a topic discussed amongst their direct community, with the suggestion that their own knowledge of AMR was influenced by this.

However, the post-musical focus groups suggested that the musical had stimulated discussion around these topics at home and wider. Furthermore, after the musical children were able to identify ways they might be able to involve themselves in this process of engaging with others on a personal and educational level. They showed intention to spread the message of AMR to others (Table 2). This suggests that the musical initiated dialogue around antibiotics and AMR in a form that also dissipated the knowledge obtained during the process to the performing child’s direct community.

#### Unique learning medium

Children and teachers felt that the musical was a fun and engaging way to learn about these topics compared to traditional teaching methods, with the messages in the musical being further reinforced by unique performance spaces. Teachers did identify time restrictions and curriculum pressures as limitations to running the musical in their schools, though recognised the potential of the musical when used as a central project around which other curriculum work could be structured (Table 3). All groups felt that there were positive psychosocial benefits to participation such as enjoyment, increased confidence and self-esteem. Children felt proud of their performances (Table 2).

**Table 3:** Feedback from class teachers pre- and two weeks post-musical.

Pre-musical	Post-musical
*‘ I think it’s great for confidence*, *and it’s great for self-esteem…’*	*‘There’s art*, *there’s literacy*, *there’s science*, *drama*, *there’s history…’*
	*‘ Honestly*, *schools are not interested in doing anything extra*. *Anything that’s going to take too much time away*.*’*
	*‘… we were all a bit like*, *why are we doing this*, *this is going to affect our data which is ultimately what we’re all measured on*. *But it worked*, *it did work–’*
	*‘I think like almost unconsciously the lyrics have gone in*. *I found myself doing that*, *I had a flick through the questionnaire to have a look at the questions and I found myself*, *oh that links to a song’*

## Discussion

Participating in the musical ‘The Mould that Changed the World’ resulted in a significant improvement in children’s knowledge around the key AMR messages of the musical that was sustained over time. Overall, following the musical qualitative data suggested a change in attitude towards antibiotics, with children demonstrating improved understanding of ways they could change their healthcare behaviours in preventing inappropriate antibiotic use. The musical engaged children with the concept of only using antibiotics when you need them in a way that led to children expressing motivation to spread this message further through education and their personal social influence. Children identified the musical as an effective and enjoyable way to learn about these topics.

Despite widespread familiarity with antibiotics, understanding of AMR and its implications amongst the general public is limited [7, 28] and there is a need to stimulate behaviour change. The COM-B model states that three components drive behaviour change: capability (TDF domains I, II, XIV), opportunity (TDF domains XI, XII) and motivation (TDF domains III, IV, V, VI, VII, XIII); all of these have been demonstrated in the evaluation of this intervention (Tables 2 and S2) [27].

Capability is attained through capacity to engage in the behaviour change activity and includes having the necessary knowledge and skills. The greatest increase in knowledge surrounded the key messages of the musical which addressed the ability for bacteria to become resistant to antibiotics, the risk of drug-resistant infections and the need for prudent antibiotic use. There was also a smaller but significant increase in knowledge surrounding clinical need for antibiotics despite the age of the children and the complexity of the concepts underlying this. Some other infectious diseases concepts addressed by the musical improved significantly, particularly the children’s knowledge that “not all bacteria cause disease” and that “antibiotics can disrupt the bacterial ecosystem in your body”. These are pertinent knowledge points in antimicrobial stewardship within the context of a cultural shift towards understanding and protecting the human microbiome [29]. Overall, the children’s acquired knowledge further allowed appreciation of how healthcare behaviours, such as the use of antibiotics, contribute to AMR.

Interestingly there were some aspects of knowledge in the quantitative results which did not significantly improve following the musical, namely that antibiotics are not effective against viruses and that antibiotics require a doctor's prescription (Table 1). These topics were addressed in the online learning resources provided to the schools, however teachers anecdotally reported minimal to no use of these resources due to the previously discussed time constraints (Table 3). The need to frame these concepts within the lyrics of the musical itself is valuable information for future implementation of the musical.

The musical also emphasised the relevance of AMR to each individual, identified previously as a challenge in addressing these topics [7]. This, alongside the recognition of the need for appropriate antibiotic behaviours on a personal level, emotionally engaged the children with antimicrobial stewardship. This was reflected in a change in the language used by children in the qualitative evaluation from vague statements such as implying technology may overcome AMR to specific examples around not using antibiotics for viruses. Furthermore, this was demonstrated in the way the children discussed their own behaviours after the musical with a shift towards individual empowerment. This emotional engagement alongside clear change in conscious decision-making directing behaviour also reflects the motivation component of the COM-B model [27].

Previous community interventions directed at parents and clinicians have been shown to change parental knowledge and awareness of antibiotics and antimicrobial awareness [30]. The improvement in knowledge seen in this study is also consistent with previous evaluation which identified interventions targeting schoolchildren and parents together as showing notable potential and resulting in a significant effect on short-term increase in knowledge [31]. The musical adds further to this by showing retained improvements in knowledge over a longer period and demonstrating motivation to change behaviour.

The musical itself also stimulated discussion around health, antibiotics and AMR. Before the musical children generally had limited understanding of antibiotics and AMR and indicated that these topics were not discussed in the home. After the musical children report more dialogue around this with family, thus these topics were also introduced to the non-participating direct community of the child. This suggests the potential for the musical to have an impact on a wider group than participants alone through social diffusion of knowledge and key messages: the opportunity component of the COM-B model [27]. Episodes of illness and contact with healthcare professionals will provide future opportunities for these individuals to enact change in behaviour.

Public initiatives to promote the appropriate use of antibiotics are widespread and multifaceted campaigns are thought to have the greatest influence [4]. Similarly, studies of drug prevention programmes have found that the effect of school programmes can be increased significantly by adding community interventions, such as mass-media campaigns [32]. As such, the effects of both the musical and wider existing initiatives, such as the European Antibiotic Awareness Day [5], may be strengthened when run together in parallel by building and improving existing knowledge and awareness, providing consistency of message and initiating further social dialogue. Other complementary activities include interventions for school aged children on infection delivered primarily through educational resource packs, including the Europe-wide e-Bug, which have been shown to improve short-term knowledge of infection and antibiotic use [9, 33–35].

The use of musical theatre is a novel tool in the field of AMR. There is some evidence that live theatre performances can be an effective method of delivering education in other fields such as smoking cessation and healthy eating [36, 37]. A study in HIV/AIDS education comparing provision of written material alone to participation in drama workshops found improvement in knowledge in both groups, however those participating in the drama also showed significant improvements in attitude [38]. The superiority of interactive methods of delivering drug-prevention programmes has been highlighted in a previous meta-analysis, likely through providing opportunities to exchange ideas and develop interpersonal skills [39]. This musical takes these principles and applies them to the field of antimicrobial awareness.

This study itself was limited by only being able to complete the follow up quantitative questionnaire at six months at one site. The qualitative data methodology carries some limitations also. Due to time constraints the questioning schedules did not undergo separate pilot testing prior to use. Furthermore, using a solely deductive approach in analysis of the data can lead to restriction of the analysis by the framework. In this study we mitigated this issue by using an inductive approach first and allowing the themes to organically develop, whilst still using the framework to identify the important behavioural determinants and therefore potential areas for further intervention development. A limitation of the TDF itself is that it is an all-inclusive theory making it very difficult to test [40]. Despite these limitations, a strength of this evaluation is that it is one of the few in this area that applies theoretical frameworks to its methodology and analysis of qualitative data [27, 41]. The study design was also limited by the lack of a control arm comparing an alternative methodology with an equivalent level of engagement. In this pilot, the intervention was supported by the involvement of external music and drama specialists, whilst it is anticipated that other schools would rely instead on the additional online resources provided. Assessing the impact of using these resources alone, or an alternative control arm which compared the musical with conventional teaching methodology, was not possible within the scope of this evaluation. Finally, as with much of the literature on behaviour change and AMR, it was not possible to directly assess whether the expressed changes in knowledge, attitude and engagement translated into a change in behaviour around antimicrobial use.

The design and delivery of a musical within schools also highlighted some potential challenges such as the resources and time required to practise and perform the musical around delivering the mandatory curriculum. This was reflected in the comments made by teachers in the informal feedback sessions. In this case, the performances were supported by external music and drama specialists, though for wider implementation extensive high-quality structured teaching and performance online resources are now available. These are designed to support primary school staff to produce the musical independently, providing a holistic way to deliver multiple parts of the mandatory curriculum and allowing teachers to integrate the musical into their learning plans for the year. The data suggest that children found the musical an effective way to learn about and engage with this topic, finding the messages more memorable as a result. This is reflected in sustained gains in knowledge together with an enablement and change in attitude in the performing children.

Overall, this evaluation study demonstrates the potential effectiveness of musical theatre in antibiotic awareness as a novel device to improve knowledge, change attitudes and emotionally engage the general public through children. Alongside existing interventions, it represents a further unique and valuable tool in the fight against AMR.

## Supporting information

S1 Fig Performance of this musical maps to multiple areas of the primary school curriculum. Panel A maps the activities of the musical to the National Curriculum in England and Panel B to the Scottish Curriculum for Excellence.
(DOCX)

S2 Fig (PNG)

S1 Table Musical implementation framework with structure and timing of participant workshops and data collection. In addition, between workshops schools were able to use the online resources to carry out their own sessions to learn the lyrics, music and choreography.
(DOCX)

S2 Table Focus group (n = 29) responses from participating children pre- and two weeks post-musical, mapped to the Theoretical Domains Framework. (DOCX)

S1 File Questionnaire used to collect quantitative data pre- and post-musical. (DOCX)

S2 File Questioning schedules used to collect qualitative data pre- and post-musical through focus groups. (DOCX)
