# Supplementary material for: ‘The Mould that Changed the World’: Quantitative and qualitative evaluation of children’s knowledge and motivation for behavioural change following participation in an antimicrobial resistance musical
Source: PLoS One. 2020 Oct 29;15(10):e0240471. doi: 10.1371/journal.pone.0240471 (PMC7595328; doi:10.1371/journal.pone.0240471)
Supplement: S1 Fig — Panel A maps the activities of the musical to the National Curriculum in England and Panel B to the Scottish Curriculum for Excellence. (DOCX) [file pone.0240471.s001.docx]

**S1 Fig. Performance of this musical maps to multiple areas of the primary school curriculum**. Panel A maps the activities of the musical to the National Curriculum in England and Panel B to the Scottish Curriculum for Excellence.


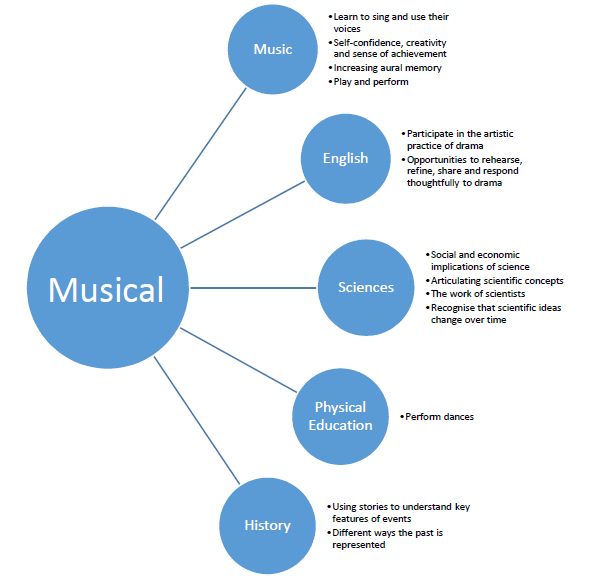

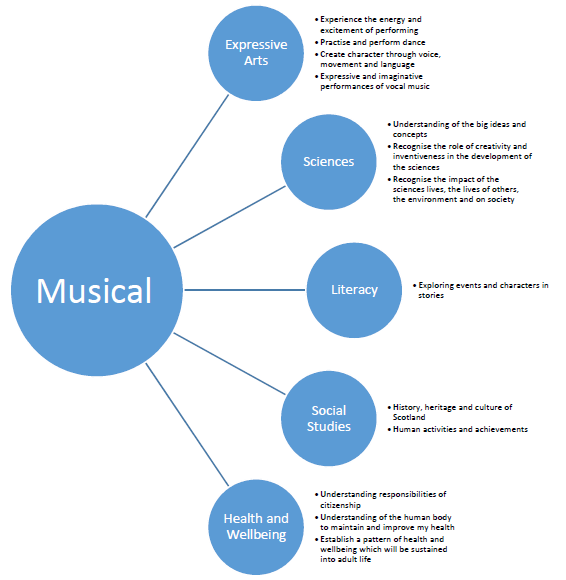


A

B
