# Supplementary figures and images for: ‘The Mould that Changed the World’: Quantitative and qualitative evaluation of children’s knowledge and motivation for behavioural change following participation in an antimicrobial resistance musical
Source: PLoS One. 2020 Oct 29;15(10):e0240471. doi: 10.1371/journal.pone.0240471 (PMC7595328; doi:10.1371/journal.pone.0240471)

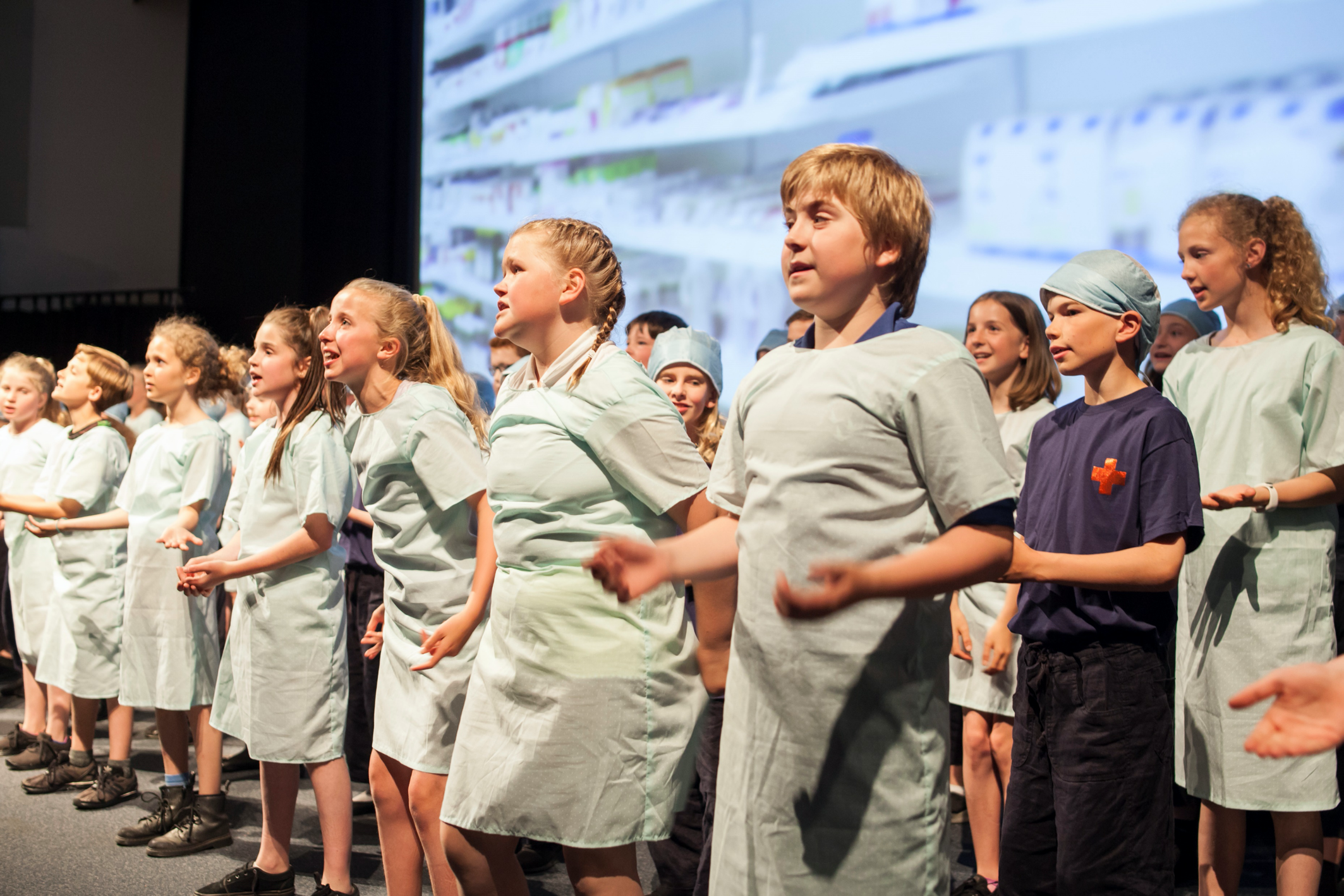

Supplement: S2 Fig — (PNG) [file pone.0240471.s002.png]
