# Supplementary material for: ‘The Mould that Changed the World’: Quantitative and qualitative evaluation of children’s knowledge and motivation for behavioural change following participation in an antimicrobial resistance musical
Source: PLoS One. 2020 Oct 29;15(10):e0240471. doi: 10.1371/journal.pone.0240471 (PMC7595328; doi:10.1371/journal.pone.0240471)
Supplement: S1 Table — In addition, between workshops schools were able to use the online resources to carry out their own sessions to learn the lyrics, music and choreography. (DOCX) [file pone.0240471.s003.docx]

**S1 Table Musical implementation framework with structure and timing of participant workshops and data collection.** In addition, between workshops schools were able to use the online resources to carry out their own sessions to learn the lyrics, music and choreography.

| **Workshop** | **Description** | **Scottish School** | **English School** |
| --- | --- | --- | --- |
| **First contact session** | - Introduction to musical for teachers - 1-2 hours total | 5/3/18 | 8/3/18 |
| **Data collection pre-musical** | - Pre-musical qualitative data collection focus groups with children carried out - Pre-musical quantitative data collection completed with children, in form of online questionnaire delivered in classroom setting | 5/3/18 | 8/3/18 |
| **Participant introductory workshop** | - First introduction to project for pupils - Interactive talk through synopsis followed by short session to start each character group on their songs - Aim to encourage enthusiasm in project and to aid teachers with task of helping children learn songs - 2-3.5 hours total | 28/3/18 | 18/4/18 |
| **Staging workshop** | - Created to teach actions and movement needed to take the show to stage - Day finishes with full run-through of show - 5.5 hours total | 24/5/18 | 18/6/18 |
| **Performance workshop** | - Opens with full cast ‘meet and greet’ followed by ‘walk through’ of show to link disparate elements and character groups. - This is followed by the dress rehearsal, with aim is to become comfortable with singing together in the performance space alongside the adult characters - Final performance run in afternoon, attended by family and friends - Total 3.5 hours plus performance time | 13/6/18 | 4/7/18 |
| **Data collection two weeks post-musical** | - Post-musical qualitative data collection focus group with children carried out - Post-musical quantitative data collection completed with children | 25/6/18 | 16/7/18 |
| **Data collection six months post-musical** | - Further post-musical quantitative data collection completed with children; Scottish school only | December 2018 | n/a |
