# Supplementary material for: ‘The Mould that Changed the World’: Quantitative and qualitative evaluation of children’s knowledge and motivation for behavioural change following participation in an antimicrobial resistance musical
Source: PLoS One. 2020 Oct 29;15(10):e0240471. doi: 10.1371/journal.pone.0240471 (PMC7595328; doi:10.1371/journal.pone.0240471)
Supplement: S2 Table — (DOCX) [file pone.0240471.s004.docx]

**S2 Table. Focus group (n=29) responses from participating children pre- and two weeks post-musical, mapped to the Theoretical Domains Framework.**

| **Domain of Theoretical Domains Framework** |  | **1. Enhanced learning and knowledge of antibiotics and AMR** | **2. Behaviour change in relation to infectious diseases and antibiotics** | **3. Engagement of individuals to spread the message of AMR** |
| --- | --- | --- | --- | --- |
| **I. Knowledge** | **Pre-Musical** | **There is widespread belief that antibiotics can treat a viral infection**  *Child: I know antibiotics is a medicine for viruses that you have.*  **Children sometimes thought antibiotics could treat conditions other than infections**  *Child: I’m thinking that, so my mum has anger issues, and I think she takes these anti, antibiotics really to calm herself down.*  **Parents generally had a good understanding of the role of antibiotics**  *Parent: It’s going to be targeting bacterial infections, not fungal, not viral.*  **A few children understood the concept of AMR**  *Child: Is it like, where bacteria gets too strong or something, and the antibiotics can't help it?*  **More commonly, children thought AMR was due to the body getting ‘used to’ antibiotics**  *Child: Then won’t be able to take them anymore because then you’ll be resistant to them.*  **Generally parents had a more sophisticated understanding of the development of AMR than children**  *Parent: So obviously then it hasn’t really wiped out the bacteria, so that you will then, the bacteria will come back and the ones that have developed a little bit of resistance will come back stronger and stronger.* | **Some children avoid antibiotics because they perceive them as addictive**  *Child 1: …if you take more antibiotics than you need …*  *Child 2: Might get addicted.* | **Some thought that if antibiotics work now, there is no reason that they won’t work in the future**  *Interviewer: So then do you think it’s not going to be a problem, we don’t need to worry about antibiotic resistance?*  *Child 1: I don’t think so, no.*  *Interviewer: You don’t think so.*  *Child 1: Because if it works now there’s no reason why it wouldn’t work …*  *Child 2: In the future.* |
|  | **Post-Musical** | **Some children still did not know what antibiotics do**  *Child: Wait, is antibiotics painkillers?*  **Others demonstrated improved understanding of what antibiotics do**  *Child: Don’t use antibiotics when you have a cold.*  *Interviewer: Any times when you might not use antibiotics?*  *Child 1: When it’s not that serious.*  *Child 2: Yeah if you’re using it for colds and things, like flu.*  **Most children had a better understanding of AMR**  *Child: Because bacteria evolved to not, so that, they can’t, the antibiotics can’t stop them.* | **Children and parents knew that antibiotic overuse was key in the development of AMR**  *Child: My takeaway is that we shouldn’t be using the antibiotics when we don’t need it, that’s why these bacteria, they're getting resistant to it.* | **Children recognise that changing antibiotic use is important in reducing AMR**  *Child: So I think it’s just, the whole musical is just a message to people to say that, don’t use antibiotics when you don’t need it.*  **Parents felt the musical gave them a better understanding of the scale of AMR**  *Parent: …I think I didn’t realise quite how big an issue it is, that it’s on an international scale. So I think it has increased my awareness a bit.* |
| **II. Skills** | **Pre-Musical** | Not a prominent theme | Not a prominent theme | Not a prominent theme |
|  | **Post-Musical** | Not a prominent theme | Not a prominent theme | Not a prominent theme |
| **III. Social/professional role and identity** | **Pre-Musical** | **Some children identified a role for technology and science in targeting AMR**  *Child: Yeah, I think what we can do is, I think they should come up with new medicines to experiment like the person who discovered penicillin by accident, something similar, but I think maybe technology will solve the problem.* | **Some children suggested reducing antibiotic use to reduce AMR**  *Child: Maybe we could try and like not take so many antibiotics and our body be able to fight the diseases, because then we wouldn’t need antibiotics.*  **Parents recognise conflict between their role as primary carer and a desire to reduce AMR**  *Parent: It is hard to know what to do because to be honest, even though I know there’s an issue of antibiotic resistance, I think I’d rather have had my child have antibiotics than be in pain.* | **A few children felt that there was a role for education in tackling AMR**  Child: …*it's quite educational as well because, especially when it's getting sent to child, to every child over the nation, it can help teach people, it teaches people about the simple ways of viruses and how you can cure them properly.*  **Parents identify reducing antibiotic use as a key target in AMR**  *Parent: I think personally what you can do is use them less. Don’t push to use antibiotics, and in fact less medicine is often better.*  **Parents and teachers felt education around use of antibiotics was important**  *Teacher: When they go to the doctors when they’re older, they’ll already know that antibiotics isn’t necessarily the best thing for them. So they won’t go in there asking for them, and I think if we can develop a generation of children who, when they’re adults, they don’t go and ask for antibiotics, I think that will go a long way in solving the problem, just leaving it up to the doctors.* |
|  | **Post-Musical** | **Science and technology was again identified as important in the fight against AMR**  *Child: In the future they’ll invent, probably they’ll invent something that can cure anything.*  **Children demonstrated understanding of the social factors contributing to AMR**  *Child: You shouldn’t overuse them. If we have to use them then we have to use the full dose.* | **Children felt they had a role in changing their own health behaviours**  *Child: …if like your mum says you might need to get some antibiotics you might think like maybe I actually probably don’t need that, like you could say to your mum I probably won’t need antibiotics for this.*  **Children demonstrated antimicrobial stewardship**  *Interviewer: Do you think there’s anything that you can do or that we can do to prevent antibiotic resistance?*  *Child: To not overuse it.*  *Child: Be more careful with it and just listen to your doctor’s advice.*  **Some parents felt the musical would make them question healthcare professionals who prescribed antibiotics**  *Parent: So I think it is good, and I think it would probably make me, if I’ve been given antibiotics, it would probably make me question whether I needed them.* | **Children identified a role for themselves in spreading the message of AMR**  *Interviewer: And what do you think is your role personally to make a difference to antibiotic resistance?*  *Child: To warn people who are, who you might think are using it the wrong way.*  **Parents feel there is a role for education to change attitudes around antibiotics and AMR for both patients and doctors**  *Parent: I do think every bit of education helps and I think there are, short of making people, banning the use of antibiotics or something. If you can make people more aware through education then that’s definitely not a bad thing.*  **Parents felt the musical stimulated discussion around antibiotics and AMR**  *Parent: We discussed it within the wider family as well. So, undoubtedly, my eldest child probably had some awareness before about antibiotics and their overuse, but my youngest certainly did not. So I think it’s been an incredibly powerful event for him to have been involved with, and a learning opportunity for him.*  **Parents felt there was a role for the musical in spreading awareness of AMR and have engaged in this process**  *Parent: As the Chief Medical Officer says, what’s it, health by stealth. The message has got out through the medium of song to those children in particular, and I have become quite evangelical…*  **Parents feel that spreading AMR awareness through children is a powerful tool**  *Parent: I think definitely a lot of people think that you get the message through to children and get habits formed and thought processes formed early on then that’s a good way to go.* |
| **IV. Beliefs about capabilities** | **Pre-Musical** | **A few had knowledge that some infections are self-limiting and can be treated by simple measures**  *Child: …it's very rare that a flu, a cough was all of a sudden start to kill you but there's simple cures…* | Not a prominent theme | **Teachers feel capable of introducing AMR to children in their class**  *Teacher: So if you make them aware of it before they start independently going to a doctor, then they might not necessarily go in there with the preconceived idea of, right I need antibiotics. They might go in there already thinking, I probably don’t need antibiotics, that’s probably the last port of call for me, but I do need some advice, or they might even not go to the doctor and try and deal with it themselves.* |
|  | **Post-Musical** | **Increased knowledge of AMR enabled children to feel capable of playing a role in tackling this**  *Interviewer: Do you think there’s anything that you can do or that we can do to prevent antibiotic resistance?*  *Child: To not overuse it.*  *Child: Be more careful with it and just listen to your doctor’s advice.* | **Children felt capable of reducing antibiotic use**  *Interviewer: Do you think that there’s anything that we can do or that you can do as an individual to help prevent antibiotic resistance?*  *Child: Don’t take them if you don’t need them.*  **Many children felt capable of caring for themselves when feeling unwell, without using antibiotics**  *Child: Orange juice.*  *Child: Lozenges.*  *Child: Yeah.*  *Child: Vitamin C or something like that instead of having antibiotics.* | **Children believe they are capable of changing AMR through educating others**  *Interviewer: So what's your role then, now?*  *Child: Spread the word.*  *Children: (general agreement)*  **Parents feel that it is possible for society to prevent AMR by working together**  *Parent: But what I do think about is the fact that we’ve had really big issues before. We had a big problem with the ozone layer, and we stopped using certain chemicals, and that seemed to have an impact. So I think if we are aware of things and take action it does seem to help.* |
| **V. Optimism** | **Pre-Musical** | Not a prominent theme | Not a prominent theme. | **Some children felt optimistic that the musical would help others to learn**  *Child: I think it will definitely spread what's been happening and awareness.*  **Some parents felt optimistic that AMR won’t ever become a big problem**  *Parent: I think that personally I have a maybe not quite rational faith that people will continue to find new treatments with the research, although the evidence points against it but somehow deep down I think we have this blind faith in the scientific research will continue, and that you won't really reach the point where the antibiotics on hand are not useful.* |
|  | **Post-Musical** | Not a prominent theme | **Children reported some optimism that their own antibiotic use would change in the future**  *Interviewer: How optimistic are you that this musical can improve the way that you use antibiotics in the future?*  *Child: Kind of optimistic.* | **Children felt optimistic that the musical could spread awareness of AMR**  *Interviewer: And how optimistic are you that this musical can change antibiotic resistance?*  *Children: (general agreement that it can)*  *Child: I think it’s a very severe message and it shows the effect on all of the infections resisting to the drugs, and so I think it’s going to help with antibiotic resistance and not using it so much.* |
| **VI. Beliefs about consequences** | **Pre-Musical** | **A few children, parents and teachers think the consequences of AMR could be serious**  *Child: … you never know, we might not find a better medicine in the future and if we just don't take care or we just leave antibiotics, more people might just start dying.*  **Many more children think AMR won’t be a problem**  *Child 1: They’ll be something newer and better.*  *Child 2: Yeah they might try to think of a new way to try and create a new medicine using the ones that you already have or just make a new one.* | **Some children don’t think AMR will affect them because they just won’t take antibiotics**  *Child: I probably wouldn’t have antibiotics anyway because every time I have a swallow pill I always chew it up and it’s really disgusting*. | **Some parents feel that AMR may be overcome before it becomes an issue**  *Parent: I think, no I know, I think we’ve, I think we’ve created, as a race, the human race has created a lot of problems for itself in the past and solved them. So, it’s, maybe we’ll do that again and I think part, I think there, people are already finding that they don’t always have to use antibiotics.*  **Parents felt the musical would be an effective way to spread education about AMR**  *Parent: I think putting on a show to friends and family will be really positive. I hope that they all learn something out of it and become a little bit belligerent in passing the message on to other people about the, about antibiotics and the fact that we do have to be careful about their use and only have them when we need them.*  **Some teachers feel that education has the potential to impact the future of AMR**  *Interviewer: What do you think we can do to prevent antibiotic resistance?*  *Teacher: On a scientific level, I have no idea. Just as a general citizen, I think that not relying on them as a cure for everything would be a good starting place, and I certainly think education has a big part to play in that.* |
|  | **Post-Musical** | **Some children still had misconceptions about AMR**  *Child: I’ve never taken antibiotics so it’s not going to affect me.*  **Children identified potentially devastating consequences of AMR**  *Child: Everybody will die, the world, sorry --*  *Child: Loads of people will probably die of infection or other stuff.*  **Parents felt the consequences of taking part were greater than simply watching the musical**  *Parent: I’m not, I have no idea about other children watching such a performance, but certainly participating in it, they got it.* | **Children believed the musical could change the behaviour of others**  *Child: Somebody in the audience is using antibiotics, they might use it properly.*  **Parents believe that the musical will influence attitudes and behaviours**  *Parent: I think definitely a lot of people think that you get the message through to children and get habits formed and thought processes formed early on then that’s a good way to go.*  **Some parents believe the musical will have an impact on their own attitude and behaviour**  *Parent: No, I think it’s made me, it’s reminded me about antibiotics I think probably. So I think it is good, and I think it would probably make me, if I’ve been given antibiotics, it would probably make me question whether I needed them.* | **Many children felt the musical could spread the message of AMR to others to make an impact**  *Child: And so my family really never, we never used to talk about it, never used to think about it, and so I think when people will see this play or hear about it or the message which is coming through, I think it’s important that the people who are watching the play, that they get the message as well, so that it can spread. And I think if that happens maybe it might make a difference.*  **Parents also recognised a positive impact on others who were not participating**  *Parent: And even my other child, who’s not involved in the production and who is at high school, was able to reel off the lyrics for a song, just the other day actually, and did it word perfect. And he wasn’t even at the performance, so yes, it had quite an impact upon my children, certainly.* |
| **VII. Reinforcement** | **Pre-Musical** | Not a prominent theme | **Children try to avoid antibiotics because they taste unpleasant**  *Child: When I was on penicillin my, even though I really hated it because it tasted like bike oil…* | Not a prominent theme |
|  | **Post-Musical** | Not a prominent theme | **Some children just wouldn’t take antibiotics because they taste unpleasant**  *Child: Well I’ve had antibiotics before and it kind of tastes disgusting, so I wouldn’t worry about me having it because I don’t really like it, I wouldn’t want to keep having it.* | Not a prominent theme |
| **VIII. Intentions** | **Pre-Musical** | Not a prominent theme | **Children report that they would take advice from a doctor if feeling unwell**  *Child: I don't really get unwell a lot but if I did go to the doctor I would take medicine and stuff and the doctor would tell me what to do.*  **Parents intend to seek and follow the advice of a doctor when feeling unwell**  *Parent: I definitely rely a lot on medical advice, and I tend to rely on them knowing when to use antibiotics.* | Not a prominent theme |
|  | **Post-Musical** | Not a prominent theme | **Children intend to use antibiotics more carefully in the future**  *Interviewer: Do you think it might change the way that you think about antibiotics in the future?*  *Child: Yeah, to use them much more carefully, like when you grow up you don’t just pick it because your head’s hurting.*  **Parents intend to use antibiotics appropriately and be guided by their doctor**  *Parent: I rely on professionals, so I rely on going to the doctor and if they tell me I need antibiotics I would say, that’s fine. I wouldn’t question it really, and if they say, we’re trying not to use antibiotics so you can do this, I would accept that as well.* | **Children intend to spread the message of AMR to others**  *Interviewer: And what do you think is your role personally to make a difference to antibiotic resistance?*  *Child: To warn people who are, who you might think are using it the wrong way.*  **Some parents intend to spread the word about the musical to others**  *Parent: As the Chief Medical Officer says, what’s it, health by stealth. The message has got out through the medium of song to those children in particular, and I have become quite evangelical (inaudible). I have sent on the link to a number of people about the musical...* |
| **IX. Goals** | **Pre-Musical** | Not a prominent theme | Not a prominent theme | Not a prominent theme |
|  | **Post-Musical** | Not a prominent theme | Not a prominent theme | **Some children want to spread the message further afield**  *Child: Yeah, and if it’s, we start in the UK and then I think if we did it in different countries --*  *Children: (general agreement)*  *Child: That would be amazing.*  *Child: If it does, because then all of this is the United Nations, and I think maybe if it somehow does get, if the United Nations do hear about it, I think they might make a change because I think they might think about it more.* |
| **X. Memory, attention and decision processes** | **Pre-Musical** | Not a prominent theme | **There was a strong social influence on children’s healthcare behaviour**  *Child: Well, my, I normally go to my parents first and tell them I feel unwell.* | **Despite occupational exposure to AMR, not all parents had considered the consequences of AMR**  *Interviewer: In terms of, does anyone ever talk about the future? So, what might happen if we run, if resistance becomes much more widespread --*  *Parent: Yeah.*  *Interviewer: And we can’t treat infections the same way?*  *Parent: I’ve not ever had that conversation actually in my work environment or socially.*  *Interviewer: Is it something you’ve thought about?*  *Parent: I do sit and listen to, on our ward rounds we have a microbiologist come along to our ward rounds and their concerns but I do still think we often treat with antibiotics. We do often and that’s but we’re, it’s more complex I think.*  **AMR doesn’t always feel relevant to the individual, which affects engagement with acting on it**  *Teacher: ... I guess that the fear isn’t real enough yet, so it’s probably not having the impact that people would like it to have, because people go, well you can still get antibiotics (inaudible) so I’m not worried about it too much.*  **Teachers feel that influencing children at this age will affect their long-term behaviour**  *Teacher: When they go to the doctors when they’re older, they’ll already know that antibiotics isn’t necessarily the best thing for them. So they won’t go in there asking for them, and I think if we can develop a generation of children who, when they’re adults, they don’t go and ask for antibiotics, I think that will go a long way in solving the problem, just leaving it up to the doctors.* |
|  | **Post-Musical** | Not a prominent theme | **Overfamiliarity with topics can lead to lack of engagement**  *Parent: Because I think you can get fatigued by thinking about these things, when you stop to think about it, so I think it’s always good to be reminded about things. No, I think it’s made me, it’s reminded me about antibiotics I think probably. So I think it is good, and I think it would probably make me, if I’ve been given antibiotics, it would probably make me question whether I needed them.* | **Parents felt that spreading the message through children was powerful**  *Parent: I think definitely a lot of people think that you get the message through to children and get habits formed and thought processes formed early on then that’s a good way to go.* |
| **XI. Environmental context and resources** | **Pre-Musical** | **Some children had prior knowledge of infections and AMR through their school**  *Child: I heard it all from our class teacher in a science lesson, and we were learning about bacteria, viruses and so yeah.* | Not a prominent theme | **Some parents feel that education around AMR is important to reduce pressure on GPs**  *Parent:I know that GPs come under an awful lot of pressure from patients to leave the surgery with some kind of prescription. And I think if there was more of an understanding about the fact that we might all suffer in the long run the more antibiotics are prescribed in inappropriate circumstances then that can only be a good thing.*  **Some parents think that we are doing will be futile if the message can’t be spread worldwide**  *Parent: I've done a lot of work in China and Southeast Asia and it really is quite mad how freewheeling people are with antibiotics, really broad spectrum antibiotics being available over the counter. I think Australian doctor friends who were with us in China, they were just flabbergasted that you can buy that. That’s one of our last lines, and people are just using it willy nilly. So I think that it would really be wise to spread the message further, if possible.* |
|  | **Post-Musical** | **Some parents believe only certain social/geographical groups have proper access to antibiotics and that certain groups are the cause of AMR**  *Parent: One of the things as well is the fact that, the idea that it’s still good to use antibiotics but use them carefully, because there’s still a lot of people that don’t have access to it, so they will be helpful. The impression I got, it’s the wealthier societies, or the wealthier parts of the world that are overusing it and there needs more education.* | **Parents recognise the tension between expectations of the public and the wider consequences of antibiotic prescribing**  *Parent: And it is about encouraging individuals to think about their need for antibiotics and it’s about supporting doctors who, in their decisions not to prescribe antibiotics. But there is a lot of work to be done, I think, within the public psyche about the purpose of antibiotics and when it’s appropriate to take them.* | Not a prominent theme |
| **XII. Social influences** | **Pre-Musical** | **Family and friends had a strong influence on whether children learned about these topics**  *Child: My brothers don’t really know about anything about antibiotics and neither do I.*  **Some children, parents and teachers had an awareness of AMR through the media**  *Parent: Although I've definitely heard of it but I wouldn’t say I'm an expert. I do know about broad spectrum antibiotics, I do know that antibiotic misuse is causing resistance because you see that in various headlines --* | **Parents played a significant role in guiding health-related behaviours**  *Interviewer: Thinking about who decides if you go to the doctor, do you decide for yourself or does someone --*  *Child 1: Your parents.*  *Child 2: Your parents, whoever looks after you.*  **Antibiotics are discussed socially in the context of healthcare access and pressures**  *Parent: Because it’s interesting because I do speak to my friends who’re not healthcare professionals and they’re, I think a lot of people still think antibiotics are, they went to their GP and I said I need an antibiotic and they almost demand that. They think that is going to make them better because I think people think that that’s the way forward, antibiotics and the doctor doesn’t know what they’re talking about and people, that people are maybe not aware of the course of infection.* | **Very few children had discussed antibiotics or AMR with their family or friends**  *Child: I didn’t really know much about it and I knew what it was but I didn’t really know much about it and I’ve not really mentioned, asked my parents about it so I don’t know if they know or not.*  **Most children would not discuss these topics with their peers**  *Interviewer: Do you think you’d ever talk to each other about it?*  *Child 1: Maybe.*  *Child 2: Very unlikely.*  *Child 3: Yeah, unlikely.*  **Some parents know about AMR through friends and family**  *Interviewer: So then moving on to antibiotic resistance is it something you are aware of and know much about?*  *Parent: Well I think it was my brother. Yes, I am aware of antibiotic resistance and I think it’s a big worry and I think my brother had been saying something to me that there hasn’t been any new antibiotics developed in recent times and I’m not sure that there’s much research going on at the moment into developing new antibiotics and I don’t know if that is right or not?* |
|  | **Post-Musical** | **Children felt their family and friends learned something from watching the musical**  *Child: My family didn’t really know that much until the show.* | **Parents were strongly influenced by doctors**  *Parent: I rely on professionals, so I rely on going to the doctor and if they tell me I need antibiotics I would say, that’s fine. I wouldn’t question it really, and if they say, we’re trying not to use antibiotics so you can do this, I would accept that as well. I think I would still do that. I just assume that the message is going through to the professions as well.* | **Children suggested the musical had stimulated discussion around AMR at home**  *Interviewer: What do your friends or family think about antibiotics or antibiotic resistance?*  *Child: My family said that it’s scary… …they think that it’s scary because they don’t, because if they’re, if the antibiotics aren’t working on these bugs that are really, really strong because they can fight off bacteria.*  **Parents felt there was a positive impact on non-participating observers also**  *Parent: And even my other child, who’s not involved in the production and who is at high school, was able to reel off the lyrics for a song, just the other day actually, and did it word perfect. And he wasn’t even at the performance, so yes, it had quite an impact upon my children, certainly.* |
| **XIII. Emotions** | **Pre-Musical** | **A minority of children felt worried about the consequences of AMR**  *Child: Yeah, I get, I get really worried, because I always get worried, because I always try to care for my health, I do my best.*  **Some parents reported feeling worried about AMR**  *Parent: Yeah it worries, it does, yeah, it does worry me, it does worry me because I’d hate it if one of my children developed an infection that couldn’t be treated, wasn’t treated properly because of these resistant or resistant antibiotics.* | Not a prominent theme | Not a prominent theme |
|  | **Post-Musical** | **Some children felt sad about AMR**  *Child: And it’s more important to add, because the drug’s been here working for so long, and it’s kind of sad to see it not working anymore.* | Not a prominent theme | **Children had mixed feelings about the future of antibiotics**  *Interviewer: Are you worried about the future of antibiotics?*  *Child: Yes.*  *Child: No.*  *Child: Yeah, kind of.*  **Spreading the message about AMR made children feel proud**  *Child: Because spreading the message, it feels like you’re a good person, like you want to make the world think, yeah.* |
| **XIV. Behavioural regulation** | **Pre-Musical** | Not a prominent theme | Not a prominent theme | Not a prominent theme |
|  | **Post-Musical** | Not a prominent theme | Not a prominent theme | Not a prominent theme |
