# Supplementary material for: ‘The Mould that Changed the World’: Quantitative and qualitative evaluation of children’s knowledge and motivation for behavioural change following participation in an antimicrobial resistance musical
Source: PLoS One. 2020 Oct 29;15(10):e0240471. doi: 10.1371/journal.pone.0240471 (PMC7595328; doi:10.1371/journal.pone.0240471)
Supplement: S1 File — (DOCX) [file pone.0240471.s005.docx]

**S1 File: Questionnaire used to collect quantitative data pre- and post-musical**

Questionnaire for participating children

1. Pick the statement which best describes you
   1. I am in P5 at Gullane Primary school
   2. I am in P6 at Gullane Primary school
   3. I am in P7 at Gullane Primary school
   4. I am a teacher at Gullane Primary school
   5. I am a parent at Gullane Primary school
   6. I am in Year 6 at Hitherfield Primary School
   7. I am a teacher at Hitherfield Primary school
   8. I am a parent at Hitherfield Primary school
2. How many times have you been prescribed antibiotics in your life? (pick one)
   - - 1. <1
       2. 1-5
       3. 5-10
       4. >10
3. I have learnt about antibiotic resistance (pick any that apply)
   - - 1. Never
       2. In the news
       3. From teachers
       4. From parents
       5. From the musical
4. Infectious disease (True/False)
   1. Can be caused by bacteria and viruses T
   2. Infectious disease can be spread from person to person T
   3. Hand washing dose not help in reducing transmission of infectious diseases F
   4. Certain infections can be prevented by vaccines T
   5. Infections can only get better if treated with antibiotics F
   6. Self-limiting infections can be fought by the body’s natural defences alone T
5. Bacteria (True/False)
   1. All bacteria cause disease F
   2. Bacteria can live naturally on/in your body and in the environment T
   3. Bacteria can become resistant to antibiotics T
   4. Antibiotic resistant bacteria cannot spread from person to person F
   5. Bacteria can pass the ability to be resistant to other bacteria by sharing genetic information T
6. Antibiotics (True/False)
   1. Antibiotics were invented less than 100 years ago T
   2. The invention of antibiotics has shortened the average duration of life F
   3. Antibiotics are essential for treating serious bacterial infections T
   4. Antibiotics only kill bad bacteria F
   5. Antibiotics can prevent an infection developing following major surgery T
   6. Antibiotics can kill viruses F
   7. Antibiotics can disrupt the bacterial ecosystem in your body T
   8. Antibiotics have no side –effects F
   9. You can take antibiotics without a doctor’s prescription F
   10. Antibiotics are cheap and easy to invent F
7. Antibiotic-resistant infections (True/False)
   1. Antibiotic resistant infections can be life-threatening T
   2. Antibiotic resistance means you are resistant to the antibiotics F
   3. Anyone can suffer from an antibiotic resistant infection T
   4. The number of antibiotic resistant infections in the world is increased by careful, prudent use of antibiotics F
8. If I become ill… (True/False)
   1. If I get a headache and runny nose I will definitely need antibiotics F
   2. If I get a fever it is possible to get better without antibiotics. T
   3. If I am not given antibiotics by the doctor, this means the doctor does not believe I am sick F

Post- musical additional questions (Likert scale)

| I found the musical easy to understand |
| --- |
| The musical has changed the way I view antibiotics |
| We looked at the online learning exercises in class |
| I enjoyed the rehearsals with Charades theatre company |
| I enjoyed the experience of performing |
| Tell us anything you feel you would like to about the musical |
