# Supplementary material for: ‘The Mould that Changed the World’: Quantitative and qualitative evaluation of children’s knowledge and motivation for behavioural change following participation in an antimicrobial resistance musical
Source: PLoS One. 2020 Oct 29;15(10):e0240471. doi: 10.1371/journal.pone.0240471 (PMC7595328; doi:10.1371/journal.pone.0240471)
Supplement: S2 File — (DOCX) [file pone.0240471.s006.docx]

**S2 File. Questioning schedules used to collect qualitative data pre- and post-musical through focus groups**

# School childrens’ focus group schedule – Pre musical

## Introduction [please read this to the group before the focus group takes place]

My name is **X**, I work for **X** – I am conducting this focus group on behalf of **X** as part of a study to evaluate the “The Mould that Changed the World” musical. These focus groups will be used to help us understand school children’s thoughts and opinions on the topics covered in the musical before they take part in it.

If you don’t mind, the focus group will be recorded and I will take a few notes.The notes and recording will be anonymised before we type it up, meaning we will not use your name or any other information that could be used to identify you. Are you happy to go ahead with the focus group? Can I check that you have signed the consent form?

#### Background Questions

1. Can everyone introduce themselves for the purpose of the recording?
   1. Name and age.
2. Who here has heard of “The Mould that Changed the World” musical?
   1. If so, what do you know about it?

#### Section 1: Antibiotics and common infections

I’m just going to start by asking you a few questions around antibiotics and common infections…

1. What do you know about antibiotics?
   1. Probe: what they are, used for etc. *(Knowledge)*
2. What do you know about common infections like colds, runny noses, sore throats etc?
   1. Probe: type of infections (bacterial, viral), how to treat them etc. *(Knowledge)*
3. What do you think you can do to stop infections from spreading? *(Knowledge)*
   1. Probe: Wash hands regularly and use tissues to catch coughs and sneezes
4. Can anyone explain what antibiotic resistance is? *(Knowledge)*
5. Have you learnt about antibiotics, infections and antibiotic resistance anywhere else? *(Environmental context and resources)*
   1. Probe: Teachers, parents, the news etc.
6. What do your friends and family think about using antibiotics? *(Social influence)*
7. If you want to know more about these topics where would you look, or who would you ask for information? *(Environmental context and resources)*
8. What do you do when you’re unwell e.g. a cold, sore throat, runny nose? *(Skills)*
   1. Probe: Go to the doctors? Stay at home and rest? Take paracetamol?
9. Where do you go to for help when you’re unwell? *(Memory, attention and decision making)*
   1. Probe: doctors, pharmacist, family member, internet, no one.
10. How do you decide whether or not to go to the doctor’s when you’re ill? *(Memory, attention and decision making)*
11. Under what circumstances might you go to the doctors specifically for antibiotics? *(Intentions)*
    1. Probe: Severity of illness, told to by a parent or guardian, they’ve worked before
12. Is it important to you to try and conserve antibiotics? *(Goals)*
13. What do you think will happen in the future if antibiotics don’t work? *(Beliefs about consequences)*
14. How does the issue of antibiotic resistance make you feel? *(Emotion)*
    1. Probe: worried, scared, re-assured, nothing?
15. What do your friends and family think about antibiotic resistance? If anything. *(Social influence)*
16. Do you think there is anything you can do to prevent antibiotic resistance? If so, what can you do? *(Professional role and identity)*

#### Section 2: The musical

Now I want to ask you your thoughts about the musical “The Mould that Changed the World”…

1. How confident do you feel about the prospect of taking part in the musical? *(Beliefs about capabilities)*
   1. Probe: Is there anything that you’re worried about?
2. What do you think the benefits might be of taking part in or watching this musical? *(Beliefs about consequences)*
3. Do you think there might be any disadvantages? *(Beliefs about consequences)*
   1. Probe: feeling more informed, uncool to take part,

# School childrens’ focus group schedule – Post musical

## Introduction [please read this to the group before the focus group takes place]

My name is **X**, I work for **X** – I am conducting this focus group on behalf of **X** as part of a study to explore attitudes and experiences to “The Mould that Changed the World” musical. The focus groups will be used to help us inform how we implement and develop the musical for use in schools across the country.

If you don’t mind, the focus group will be recorded and I will take a few notes.The notes and recording will be anonymised before we type it up, meaning we will not use your name or any other information that could be used to identify you. Are you happy to go ahead with the focus group? Can I check that you have signed the consent form?

## Background Questions

1. Can everyone introduce themselves for the purpose of the recording?
   1. Name and age
2. Can you tell me about when you saw/took part in the musical?
3. What were your roles in the musical? (Whether active or just an audience member)
4. What were your initial thoughts of the musical?
5. Can you tell me what you remember from the musical? (Memory, attention and decision processes)
6. How well do you remember the messages from the musical? (Memory, attention and decision processes)
7. How effective was the musical in helping you remember these topics? (Memory, attention and decision processes) Probe: Compared to a normal lesson
8. Were you able to take part in the musical? (Skills)
9. Are there any reasons why you couldn’t take part in the musical? (Skills)
10. Did you struggle with any parts of the musical? Probe: Terminology, ease of script, directions, singing, remembering words (Skills)
11. What if anything, has the musical taught you about antibiotics? (Knowledge)
12. What if anything, has the musical taught you about infections? (Knowledge)
13. What have you learnt about antibiotic resistance? (Knowledge)
14. What have you learnt about looking after antibiotics? (Knowledge)
15. Was there anything confusing or you didn’t understand in the musical? (Knowledge)
16. How confident would you feel about doing another similar musical? (Beliefs about capabilities)
17. How easy or difficult has it been to be part of the musical and do your usual school work? (Environmental Context and Resources)
18. What do you think are the benefits of taking part or watching this musical? (Beliefs about consequences)
19. Do you think there are any disadvantages? (Beliefs about consequences) Probe: feeling more informed, misunderstanding
20. How important is it for other children in other schools to have this musical? (Goals)
21. How likely are you to discuss the musical with your friends and family? (Intentions)
22. How did watching/acting in the musical make you feel? (Emotion) Probe: worried, scared, re-assured, nothing, confident?
23. Have you learnt about these topics anywhere else? (antibiotics, infections, resistance etc) (Environmental Context and Resources)
24. Have your teachers encouraged you to learn about these topics? Have you learnt about it in class? (Environmental Context and Resources)
25. How do you think this musical could impact on antibiotic resistance? (Beliefs about consequences)
26. How optimistic are you that this musical can prevent antibiotic resistance? (Optimism)
27. How optimistic are you that this musical can improve the way school pupils use antibiotics? (Optimism)
28. When you think about antibiotic resistance, how does it make you feel? (Emotion)
29. Do you think you have a role in promoting the messages from the musical? (Professional role and identity)
30. How confident are you that you can make a difference to antibiotic resistance? (Beliefs about capabilities) Probe: by only using antibiotics when they’re needed, by promoting this message.
31. Do you think you have a role in preventing antibiotic resistance? If so, what can you do? (Professional role and identity) Probe: Tell friends and family about these messages, only use antibiotics when they’re needed
32. How important is it to you to try and keep antibiotics working? (Goals)
33. How important is it to you to try and stop antibiotic resistance? (Goals)
34. What do you think will happen in the future if antibiotics don’t work? (Beliefs about consequences)
35. What do your friends and family think about using antibiotics? (Social influence)
36. What do your friends and family think about antibiotic resistance? (Social influence) Probe: Do they know about it?
37. Is there anything you will do differently now as a result of the musical? (Intentions) Probe: self-care for common infections
